# The Pseudotyped Replication-Deficient VSV with Spike from PEDV Induces Neutralizing Antibody Against PEDV

**DOI:** 10.3390/vaccines13030223

**Published:** 2025-02-24

**Authors:** Jingxuan Yi, Huaye Luo, Kang Zhang, Lilei Lv, Siqi Li, Yifeng Jiang, Yanjun Zhou, Zuzhang Wei, Changlong Liu

**Affiliations:** 1 College of Animal Science and Technology, Guangxi University, Nanning 530005, China; 2218393075@st.gxu.edu.cn (J.Y.); 2318402009@st.gxu.edu.cn (K.Z.); 2 Shanghai Veterinary Research Institute, Chinese Academy of Agricultural Sciences, Shanghai 200241, China; 821012420007@caas.cn (H.L.); 82101211645@caas.cn (L.L.); 82101231350@caas.cn (S.L.); jiangyifeng@shvri.ac.cn (Y.J.); yjzhou@shvri.ac.cn (Y.Z.); 3 Jiangsu Co-Innovation Center for the Prevention and Control of Important Animal Infectious Disease and Zoonosis, Yangzhou University, Yangzhou 225009, China

**Keywords:** porcine epidemic diarrhea virus, spike protein, vesicular stomatitis virus, pseudovirus, vaccine

## Abstract

Background: Porcine epidemic diarrhea virus (PEDV) is a significant pathogen in swine, causing substantial economic losses worldwide. Despite the availability of existing vaccines, there is a critical need for novel vaccine platforms that ensure robust protection while maintaining safety. Methods: A recombinant replication-deficient vesicular stomatitis virus (VSV) vaccine, rVSV∆G-PEDV-S, was developed by pseudotyping the virus with the spike (S) protein from PEDV. To achieve high-titer pseudotyped rVSV particles, a stable Huh7 cell line expressing the PEDV S protein (Huh7-PEDV-S) was generated. The infectivity and replication capacity of rVSV∆G-PEDV-S were evaluated in PEDV-susceptible cell lines and Huh7-PEDV-S cells. The vaccine’s immunogenicity and safety were assessed in BALB/c mice vaccinated intramuscularly with rVSV∆G-PEDV-S. Results: The pseudotyped rVSV∆G-PEDV-S demonstrated infectivity in PEDV-susceptible cell lines and robust replication in Huh7-PEDV-S cells, while remaining replication-deficient in non-complementary cells. In vaccinated BALB/c mice, the vaccine elicited a strong humoral immune response, characterized by high levels of PEDV S1-specific IgG and neutralizing antibodies. No adverse effects, including weight loss or behavioral changes, were observed in the vaccinated mice, confirming the vaccine’s safety. Conclusions: The rVSV∆G-PEDV-S vaccine represents a promising platform for controlling PEDV outbreaks. Its replication-deficient design and pseudotyping methodology ensure safety and adaptability to emerging PEDV variants. These findings highlight the potential of rVSV∆G-PEDV-S as a safe and effective solution to the ongoing challenges posed by PEDV.

## 1. Introduction

Porcine epidemic diarrhea (PED) is an acute, highly contagious intestinal disease of swine caused by porcine epidemic diarrhea virus (PEDV) [1]. The virus was first identified in the United Kingdom in 1971. The G1a subtype strain has been endemic in China since 1984, and the CV777 vaccine was effective in controlling PED outbreaks. However, in 2010, a highly pathogenic G2b subtype variant strain caused outbreaks in several Chinese provinces [2] and later caused a pandemic in the United States in 2013 that spread to Canada and Mexico [3]. PED has caused great losses to the swine industry worldwide.

PEDV, a member of the Coronaviridae family, is a single-stranded positive sense RNA virus with a genome size of approximately 28 kb and a surface-modified S glycoprotein [1,4]. The S protein is a highly glycosylated type I membrane protein consisting of two distinct subunits, S1 and S2. The surface unit, S1, binds to the receptor while the transmembrane unit, S2, facilitates fusion of the virus with the cell membranes. Therefore, S proteins are key proteins for the successful infection of host cells by viruses, as well as being the main targets of neutralizing antibodies and triggering CD4+ and CD8+ T cell responses [5]. Therefore, S proteins are the main antigens for the study of targeted vaccines against PEDV.

At present, there is no targeted specific drug for PEDV, so the development of PEDV vaccines is important for controlling the spread of PEDV and the occurrence of epidemics [6,7,8,9,10,11,12,13,14]. The current application of PEDV vaccines mainly includes inactivated and live attenuated vaccines. The inactivated vaccine has high safety but a poor protective effect; the attenuated vaccine has good immunogenicity, but there is a risk of virulence reintroduction. In addition to the two traditional vaccines, a series of other vaccines, such as subunit vaccines, recombinant viral live vector vaccines, recombinant bacterial live vector vaccines, and nucleic acid vaccines, have also achieved certain research results [15]. Among them, the PEDV S protein expressed by VSV viral vectors has shown potential for use in developing vaccines against PEDV [16].

Vesicular stomatitis virus (VSV), a member of the *Rhabdoviridae* family, is a non-segmented, single-stranded, negative-sense RNA virus that encodes five structural proteins, namely nucleoprotein (N), phosphoprotein (P), matrix protein (M), glycoprotein (G), and RNA polymerase protein (L). The recombinant VSV (rVSV) platform was developed by John Rose and Michael Whitt [17,18] and has been developed as a vaccine platform for various viral pathogens, including Ebola virus (EBOV), human immunodeficiency virus, Crimean–Congo hemorrhagic fever virus, SARS-CoV-2, JEV, and Zika virus [19,20,21,22,23,24]. The rVSV platform offers several advantages, including (1) ease of proliferation and high titers; (2) strong cellular and humoral immunity in vivo; (3) attenuation of the virus by removing the VSV-G protein and reducing reactogenicity; and (4) sensitivity to IFN-α/β, which may limit replication due to an intact innate immune response [25].

The need for a safe and effective PEDV vaccine is urgent due to the continuous emergence of PEDV variants [1,2,3,15,26,27,28,29,30,31]. To address the lack of an effective PEDV vaccine, in this work, we created a replication-deficient infectious clone, rVSVΔG, which encodes the VSV-N, P, M, and L proteins and is deficient in the G gene. rVSVΔG was able to package rVSVΔG-PDEV-S in a trans-complementary Huh7-PEDV-S cell line expressing a heterologous PEDV S protein. rVSVΔG-PDEV-S does not contain an intact viral genome and is unable to proliferate in normal tissue cells, thus providing a high degree of safety [32,33]; in addition, as a live viral vector, it can induce a strong immune response in the organism [19,22,34,35,36]. Replication-defective rVSVΔG-PDEV-S may be a promising platform for the rapid development of vaccines against emerging epidemic strains of PEDV, with the promise of providing safe and effective vaccine candidates for porcine epidemic diarrhea.

## 2. Materials and Methods

### 2.1. Cell Lines, Viruses, and Antibodies

BSR-T7 (kindly provided by Prof. Xusheng Qiu), Huh7 (a gift from Prof. Rong Zhang, Fudan University), Vero (ATCC, cat: CCL-81, Manassas, VA, USA), HEK293T (ATCC, cat: CRL-3216, Manassas, VA, USA), and LLC-PK1 (MeiSenCTCC, Zhejiang, China) were cultured in DMEM containing 10% fetal bovine serum (Gibco, Shanghai, China), 100 Units/mL penicillin and 0.1 mg/mL streptomycin (Gibco, Shanghai, China). Recombinant vaccinia virus vTF-7.3 was kindly provided by Prof. Weike Li (Lanzhou Veterinary Research Institute, CAAS). The rPEDV SD-EGFP derived from PEDV SD strain (GenBank No. MZ596343) was rescued and stored in our lab. The monoclonal antibody against PEDV S protein was purchased from ZhaoRui Biotech (Shanghai, China). The HRP-linked secondary antibody for mouse IgG was purchased from Proteintech Group, Inc. (Chicago, IL, USA).

### 2.2. Plasmid Construction

The human codon-optimized full-length S gene for the PEDV SD strain was synthesized and inserted into the pLV-EF1a-IRES-Hygro (Addgene, cat: 85134, Watertown, MA, USA) vector to generate pLV-EF1a-PEDV-S-IRES-Hygro. The vector was confirmed by DNA sequencing. The cDNA clone of rVSV containing an EGFP expression cassette in place of the VSV-G gene (pVSV∆G-EGFP) was obtained from a previous study [37]. To construct the pVSV∆G vector lacking the EGFP gene, the VSV P and M genes from the pVSV∆G-EGFP expression plasmid were amplified using PCR and the EGFP gene was removed by digestion with the EcoRV restriction enzyme. After enzymatic digestion, the amplified PCR product and linearized vector were joined using the Gibson assembly method (New England Biolabs, Beijing, China). To confirm the accuracy of the recombinant plasmid, DNA sequencing was performed on the assembled product. The sequences of the PCR primers used for amplification are listed in Table 1. The helper plasmids pBS-N, pBS-P, pBS-L, and pBS-G were purchased from Kerafast (Boston, MA, USA).

### 2.3. Production and Concentration of Lentivirus

The production and concentration of lentivirus were performed as previously described [38,39]. Briefly, HEK293T cells were cotransfected using the calcium phosphate method with the lentiviral vectors pLV-EF1a-PEDV-S-IRES-Hygro, the packaging plasmid psPAX2 (Addgene, cat: 12260, Watertown, MA, USA), and the envelope plasmid pMD2. G (Addgene, cat: 12259, Watertown, MA, USA). Then, 48 h post-transfection, the virus-containing supernatants from the HEK293T cultures were clarified by centrifugation at 2000 rpm for 10 min, followed by filtration through a 0.45 μm cellulose acetate filter. The filtered viral supernatants were then concentrated using PEG6000 (Merck, cat: 25322-68-3, Shanghai, China), aliquoted, and stored at −80 °C for subsequent use.

### 2.4. Lentivirus Transduction and Cell Line Establishment

Huh7 cells were seeded at a density of 2 × 10^5^ per well in six-well plates. Twelve hours after seeding, about 5 MOIs (multiplicities of infection) of lentivirus LV-EF1a-PEDV-S-IRES-Hygro supplemented with 8 μg/mL polybrene were used to transduce the cells. At 48 after transduction, cell culture medium containing 500 μg/mL hygromycin B (Roche, cat#: 10843555001, Shanghai, China) was added. The medium was changed every 2–3 days to select for positive cells. The monoclonal cells were isolated through limited dilution in 96-well plates. The expression of the PEDV S protein was subsequently verified via Western blotting, and the best overexpressing cell lines were selected for subsequent experiments.

### 2.5. Indirect Immunofluorescence Assay

The cells were seeded in a 6-well plate at a density of 2 × 10^5^ cells per well and allowed to grow until they reached 80% confluence. Subsequently, the cells were fixed with 4% paraformaldehyde for 45 min at room temperature (RT). Permeabilization was performed using 0.25% Triton X-100 for 20 min. After washing twice with DPBS, the cells were blocked with 5% BSA for 30 min at RT. The cells were then incubated with the primary antibody at 37 °C for 1 h, followed by incubation with a secondary antibody, namely goat anti-mouse IgG (H+L) cross-adsorbed, DyLight 594 (1:1000, cat: 35511, Invitrogen, Shanghai, China) for 45 min at 37 °C. The cell nuclei were counterstained with DAPI (cat: D9542, Sigma, Shanghai, China) at RT for 5 min. Fluorescent images were captured using a BZ-X800E fluorescence microscope (Keyence, Osaka, Japan).

### 2.6. Recovery of rVSVΔG-PEDV-S

Recombinant rVSVΔG-PEDV-S was rescued using a previously established protocol [37,40,41]. BSR-T7 cells were seeded, reaching 80% confluence in T25 flasks, infected with vTF-7.3 virus at an MOI of 5 for 1 h, and then co-transfected with five plasmids: the full-length pVSVΔG (as described above), as well as the VSV helper plasmids encoding the VSV-N, P, L, and G proteins (Kerafast, Boston, MA, USA), all of which were under the control of the T7 promoter. Primary transfection was performed using Lipofectamine 3000 (Thermo Fisher, cat: L3000001, Waltham, MA, USA). Forty-eight hours after primary transfection, the supernatant containing the recovered rVSVΔG-G was collected, centrifuged at 500× *g* for 10 min to remove cellular debris, and filtered using a 0.22 µM filter to remove residual vTF-7.3 virus. For rVSVΔG-PEDV-S production, Huh7-PEDV S cells were infected with the full amount of filtered supernatant. Cells were observed daily until typical cytopathic effects (CPE) appeared and culture supernatants were collected [42,43]. The rescued viruses were initially confirmed by sequencing, and the rVSVΔG-PEDV-S virus stock was amplified by passaging in Huh7-PEDV S cells. To concentrate the recombinant VSV, the cell culture fluids was clarified by passing it through a 0.45 µM filter. The viruses were then concentrated by centrifugation at 100,000× *g* with a 20% sucrose cushion at 4 °C for 2 h in a Ti70 rotor (Beckman Coulter, Brea, CA, USA). The pelleted virions were resuspended in PBS buffer (2 mM KH_2_SO4, 137 mM NaCl, 10 mM Na_2_HSO_4_, 2.7 mM KCl 2 pH 7.4).

### 2.7. RT-qPCR for rVSVΔG-PEDV-S Titration

The RNAs of virus samples were extracted using the TIANamp Virus RNA Kit (TIANGEN, Beijing, China) according to the manufacturer’s instructions. The cDNAs were synthesized via a reverse transcription reaction with 1 µg of total RNA using the PrimeScript First-Strand cDNA Synthesis Kit (Takara, Beijing, China) [44]. These cDNAs were used for quantitative real-time PCR using SYBR Premix Ex Taq (Takara, Beijing, China) and the LightCycler 96 instrument (Roche, Shanghai, China). Ten-fold serial dilutions of the cDNA of rVSV∆G (the titer was calculated by TCID_50_) served as the standard included with each qRT-PCR assay. The viral titers were determined by interpolation onto a curve constructed of the 10-fold serial dilutions of the standards. The primers used to detect the VSV N gene sequence are shown in Table 1.

### 2.8. Western Blotting

Viruses or protein lysates were mixed with 5× loading buffer and boiled at 100 °C for 10 min before separation via 6% SDS-PAGE gel electrophoresis at 120 V for approximately 1 h. The separated proteins were transferred to a nitrocellulose membrane, which was blocked with TBST (10 mM Tris-HCl, pH 7.5, 150 mM NaCl, and 0.1% Tween-20) containing 5% non-fat dry milk at RT for 2 h. Following blocking, the membrane was incubated with mouse anti-Spike antibody (ZhaoRui Biotech, Shanghai, China) overnight at 4 °C. After washing with TBST, the membrane was incubated with HRP-coupled secondary antibody for 1 h at RT. Signals were generated using a hypersensitive ECL chemiluminescence kit (cat: P10100, NCM Biotech, Suzhou, China) and detected with a ChemiDoc™ MP imaging system (Bio-Rad, Hercules, CA, USA).

### 2.9. Animal Experiments

To identify the immunogenicity and safety of rVSVΔG-PEDV-S in vivo, we inoculated mice via the intramuscular (IM) route. As shown in Table 2, ten specific-pathogen-free 4-week-old female BALB/c mice from SPF Biotech company (Suzhou, China) were randomly divided into two groups of five mice each, namely (1) IM rVSVΔG-PEDV-S (108 TCID50/100 μL; and (2) the PBS control-vaccination group. Serum samples were collected at 0 14, 21, 28, and 42 days post-vaccination to evaluate the levels of neutralizing antibodies against PEDV SD-EGFP and specifically for PEDV IgG. In addition, to assess the safety of the vaccine, the body weights of the mice were monitored every 7 days for the first 4 weeks following vaccination.

### 2.10. Enzyme-Linked Immunosorbent Assay (ELISA)

ELISA plates were coated overnight at 4 °C with 100 ng of PEDV S1 protein per well, dissolved in coating buffer (50 mM sodium carbonate/sodium bicarbonate, pH 9.6). Following standard washing and blocking procedures, serial dilutions of serum were added in triplicate to plates and incubated for 1 h at 37 °C. After washing the plate five times with 0.05% PBS Tween 20, HRP-conjugated anti-mouse IgG was added to each well (1:5000), and the plates were incubated for an additional hour at 37 °C. Following another round of washing, color development was performed by adding 100 μL of TMB substrate to each well and incubating at 37 °C for 15 min. The reaction was subsequently terminated by adding 100 uL of 2 M H_2_SO_4_ to each well, and the absorbance was measured at 450 nm using a microplate reader (Biotek, Winooski, VT, USA). The OD_450 nm_ value of negative serum was recorded, with results classified as negative when OD_450 nm_ < $\bar{X}$ + 2SD, positive when OD_450 nm_ > $\bar{X}$ + 3SD, and doubtful when $\bar{X}$ + 2SD < OD_450 nm_ < $\bar{X}$ + 3SD.

### 2.11. Neutralization Assay

Prior to the neutralizing antibody detection, serum samples from experimental mice were heat-treated at 56 °C for 30 min, and then serially diluted 2-fold in 96-well plates containing DMEM, beginning with a dilution of 1:2. The serially diluted sera were incubated with 200 TCID_50_ per well of PEDV SD-EGFP for 1 h at 37 °C, after which a Vero cell suspension of 2 × 10^4^ per well was added. At 3 days post-inoculation, neutralizing antibody titers were determined by observing the samples under a fluorescence microscope, with the decrease in GFP positive cells indicating the presence of neutralizing antibodies. Neutralization titers were calculated as 50% inhibition of viral infection (NT_50_) using the Reed–Muench method.

### 2.12. Statistical Analyses

Data were analyzed using GraphPad Prism 9 (GraphPad, San Diego, CA, USA). Data are expressed as mean ± standard deviation (SD) of at least three replications. Statistical analysis was performed using one-way ANOVA Kruskal–Wallis test. Significance levels (*p*-values) were set at <0.01 (**), <0.001 (***), and <0.0001 (****).

## 3. Results

### 3.1. Generation and Characterization of PEDV Spike-Expressing Stable Huh7 Cell Line

**Figure 1:**
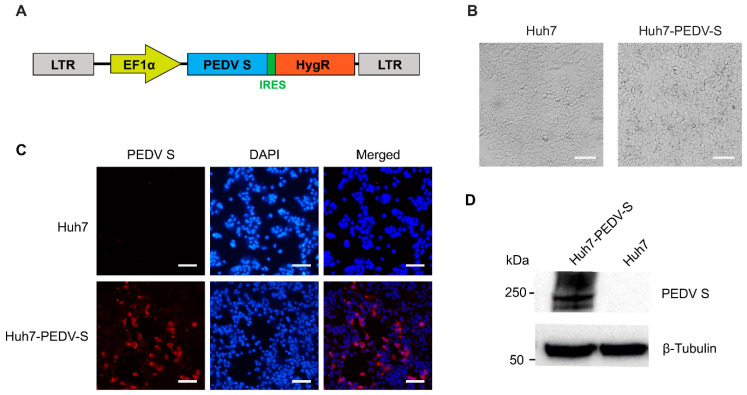
Generation and characterization of a stable Huh7 cell line expressing the PEDV spike protein. (**A**) Schematic illustration of the lentiviral vectors used for PEDV spike overexpression in Huh7 cells. LTR: long terminal repeat; EF1α: human translation elongation factor 1α; IRES: internal ribosome entry sites; HygR: hygromycin resistance gene. (**B**) Morphology of Huh7 and Huh7-PEDV-S cell lines at 10× magnification. Scale bar: 100 μm. (**C**) Representative images of immunofluorescence staining for PEDV S protein in Huh7 and Huh7-PEDV-S cell at 10× magnification. Scale bar: 100 μm. (**D**) PEDV S protein levels in Huh7 and Huh7-PEDV-S cell were determined by Western blotting.

The PEDV S glycoprotein plays a critical role in virus–host interactions and immune responses. To facilitate the trans-complementation of PEDV S pseudotyped VSV packaging, a stable Huh7 cell line expressing the PEDV S protein was developed. For this purpose, the codon-optimized spike gene was cloned into a lentiviral vector that contains a hygromycin resistance gene, linked to the spike gene via an IRES element (Figure 1A). Lentiviral particles were subsequently produced and used to infect Huh7 cells at an MOI of 5. Positive cells were selected for 7 days in the presence of 500 μg/mL hygromycin B. Single-positive cells were then manually diluted in 96-well plates. Single-cell clones were expanded and validated via Western blotting. A single clone with high expression of PEDV S was established. The Huh7 cells expressing the PEDV S protein (designated as Huh7-PEDV-S) exhibited morphology and viability comparable to that of parental Huh7 cells (Figure 1B). Immunofluorescence staining confirmed robust PEDV S protein expression in Huh7-PEDV-S cells (Figure 1C). Western blot analysis further confirmed PEDV S protein expression in Huh7-PEDV-S cells (Figure 1D). These results demonstrate that the successful generation of the Huh7-PEDV-S cell line, which stably expresses the PEDV S protein. This cell line represents a valuable tool for the production of PEDV pseudotyped VSV, enabling further research on PEDV-host interactions and pseudotyping applications.

### 3.2. Generation of Replication-Deficient PEDV S Pseudotyped Virus rVSVΔG-PEDV-S

**Figure 2:**
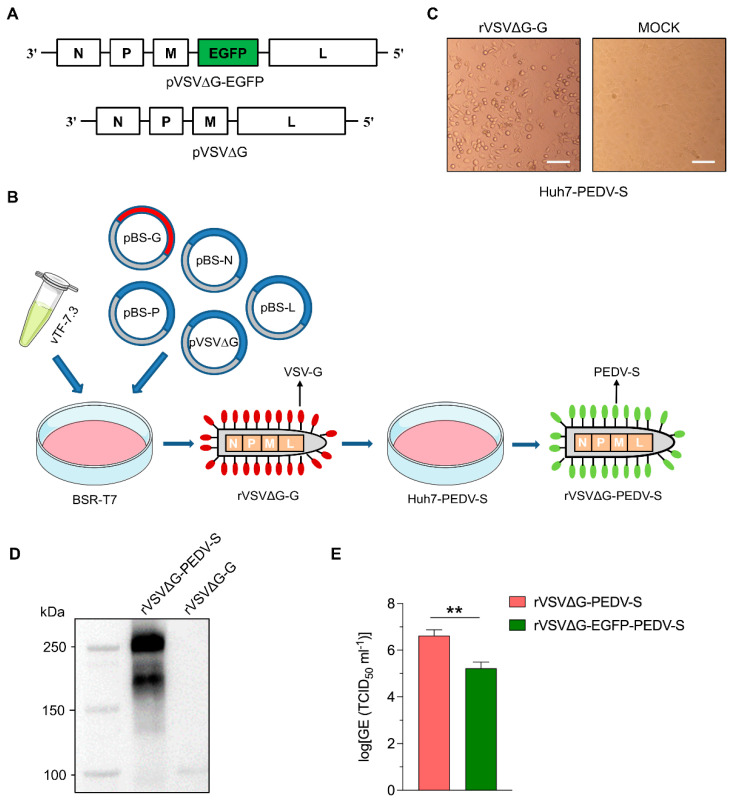
Generation of rVSVΔG-PEDV-S in the Huh7-PEDV-S cell line. (**A**) Schematic illustration of rVSV vectors used in this study. Upper panel: rVSV∆G-EGFP vector; lower panel: rVSV∆G vector. (**B**) Schematic representation of the generation process for the rVSV∆G-PEDV-S vaccine. BSR-T7 cells were infected with 1 MOI (multiplicity of infection) of vTF-7.3 virus and then co-transfected with pVSVΔG and VSV-system accessory plasmids: pBS-G, pBS-N, pBS-L, and pBS-P; Ninety-six hours after transfection, the supernatant containing rVSVΔG-G virus was collected and filtered to remove the residual vTF-7.3 virus. Subsequently, Huh7-PEDV-S cells were infected with the supernatant from the primary transfection to generate rVSV∆G-PEDV-S virus. (**C**) Morphology of Huh7-PEDV-S cells infected with the supernatant of the primary transfection or the MOCK treatment (DMEM medium) at 10× magnification. Scale bar: 100 μm. (**D**) rVSV∆G-PEDV-S virus was purified by centrifugation through 20% sucrose, and the pelleted virions were then suspended in PBS. The S protein within rVSV∆G-PEDV-S virus was analyzed by Western blotting. (**E**) The comparison of the titers of EGFP(+) and EGFP(−) rVSVs in the Huh7-PEDV-S cell line. The data are presented as log-transformed genome equivalents (GE, half-maximal tissue-culture infectious dose (TCID_50_)) per ml. values. The red bar represents rVSVΔG-PEDV-S, while the green bar represents rVSVΔG-EGFP-PEDV-S. Error bars indicate standard deviations (SDs). **, *p* < 0.01.

In our previous study, we developed a replication-deficient rVSV by replacing the G gene with an EGFP reporter gene. However, the titer of PEDV S protein-pseudotyped VSV was relatively low [37]. To improve the packaging efficiency of pseudotyped VSV with PEDV S protein, the EGFP gene was removed from the recombinant VSV genome (Figure 2A). The recombinant rVSVΔG-G virus was recovered by infecting BSR-T7 cells with 1 MOI of recombinant vaccinia virus vTF-7.3, followed by transfection with the recombinant viral vector pVSV∆G and accessory plasmids encoding VSV-N, P, L, and G proteins, all under the control of a T7 promoter (Figure 2B). Four days post-transfection, the supernatant containing rVSVΔG-G virus was collected. Residual vTF-7.3 was removed from the recovered virus via a 0.22 μm filtration and then used to infect Huh7-PEDV-S cells. Forty-eight hours post-infection, infected cells exhibited typical cytopathic effect (CPE) (Figure 2C). The supernatant containing the recovered rVSV∆G-PEDV-S was collected, centrifuged, and filtered for subsequent use. The incorporation of the spike protein was confirmed by detecting the surface protein of the PEDV pseudovirus using Western blot with a PEDV S monoclonal antibody. Specific protein bands with approximately 250 kDa were detected in the lane corresponding to the PEDV pseudovirus supernatant, whereas no corresponding bands were observed in the control (Figure 2D). To evaluate the impact of removing the EGFP gene on virus titer, we compared the titers of rVSV∆G-PEDV-S and rVSV∆G-EGFP-PEDV-S, both generated in Huh7-PEDV-S cells. The results demonstrated a significant improvement: the removal of the EGFP gene led to a ten-fold increase in viral titer (Figure 2E). This finding underscores the crucial role of optimizing the VSV genome for efficient pseudotyping and highlights the effectiveness of our approach in enhancing pseudovirus production.

### 3.3. rVSVΔG-PEDV-S Induces CPE in PEDV Susceptible Cell Lines

**Figure 3:**
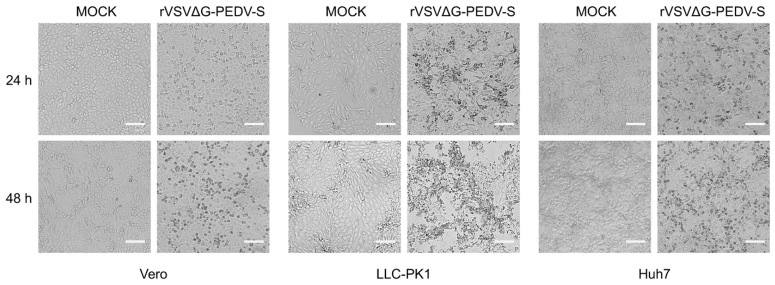
Characterization of rVSVΔG-EGFP-S entry on different cell lines. Indicated cell lines (Vero, LLC-PK1, and Huh7) were infected with equal amounts (1 MOI) of rVSVΔG-PEDV-S virus. After 24 and 48 hpi (hours post-infection), images were captured using microscopy. The representative images displayed at a magnification of 10. Scale bar: 100 μm.

To evaluate the infectivity of rVSVΔG-PEDV-S in PEDV susceptible cell lines, Vero, Huh7, and LLC-PK1 cells were exposed to equivalent amounts of the rVSVΔG-PEDV-S virus. Following infection, all three cell lines exhibited clear CPE characteristic of VSV infection. These effects included cell rounding, detachment from the culture surface, and shrinkage, which progressed to extensive cell lysis over time. The onset and severity of CPE varied among the cell lines, with Vero cells showing the most rapid and pronounced morphological changes, followed by Huh7 and LLC-PK1 cells (Figure 3). These results demonstrate that rVSVΔG-PEDV-S can efficiently enter PEDV-susceptible cell lines, inducing characteristic CPE. This highlights the utility of the PEDV S pseudotyped rVSV as a robust tool for studying PEDV entry and host cell interactions in vitro.

### 3.4. rVSV∆G-PEDV-S Could Replicate in Huh7-PEDV-S Cells

**Figure 4:**
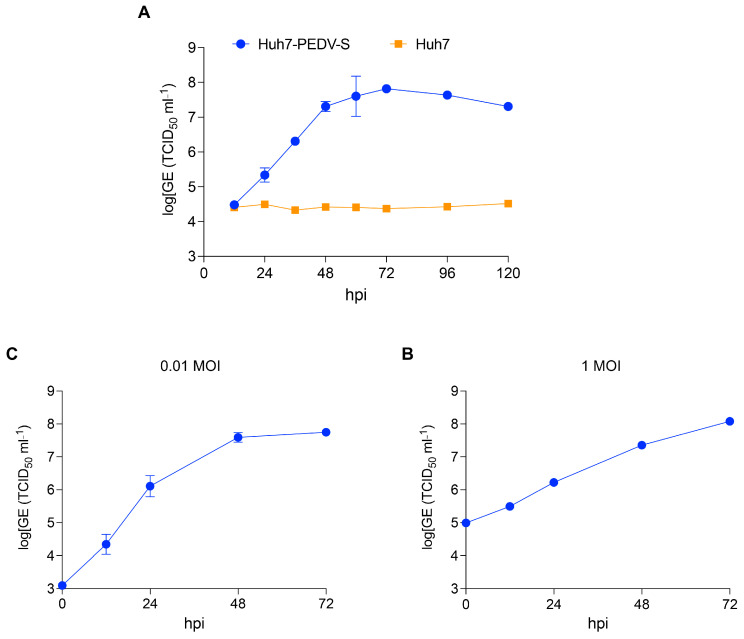
rVSVΔG-EGFP-S replicates in the Huh7-PEDV-S cell line, but not in Huh7. (**A**) Huh7-PEDV-S cells and Huh7 were infected with 0.1 MOI (multiplicity of infection) of rVSVΔG-EGFP-S virus. The levels of rVSV RNA in supernatants of the infected cells at the indicated time points after infection were quantified using a qPCR assay and expressed as genome equivalents (GEs; half-maximal tissue-culture infectious dose (TCID_50_) per mL). (**B**,**C**) Huh7-PEDV-S cells were infected with either 0.01 or 1 MOI of the rVSVΔG-PEDV-S virus. The virus titers in supernatants of the infected cells at the indicated time points post-infection were quantified using a qPCR assay. Error bars indicate standard deviations (SDs).

To investigate the replication kinetics of rVSV∆G-PEDV-S in Huh7-PEDV-S and Huh7 cells, both cell lines were infected with rVSV∆G-PEDV-S at an MOI of 0.1. The levels of rVSV RNA in supernatants of the infected cells were quantified at indicated time points post-infection using RT-qPCR. The result indicated that rVSV∆G-PEDV-S could robustly replicate in Huh7-PEDV-S. Notably, no replication of rVSV∆G-PEDV-S was detected in Huh7 cells, indicating that the presence of the PEDV S protein is essential for its replication (Figure 4A). The replication cycle of rVSV∆G-PEDV-S in Huh7-PEDV-S cells was characterized by a prolonged phase of viral amplification, culminating in high viral yields. The result further confirmed that rVSV∆G-PEDV-S is a replication-deficient virus in the absence of the complementary PEDV S protein. We also tested the rVSV∆G-PEDV-S replication at different MOIs (0.01 and 1 MOI). At an MOI of 0.01, a gradual increase in viral titers was observed, starting from 10^3.1^ TCID_50_/mL at 0 hpi and reaching a peak of 10^7.75^ TCID_50_/mL by 72 hpi (Figure 4B). Similarly, at an MOI of 1, viral titers increased steadily from 10^5^ TCID_50_/mL at 0 hpi to a maximum of 10^8.1^ TCID_50_/mL at 72 hpi (Figure 4C). These results revealed a robust replication profile of rVSV∆G-PEDV-S, demonstrating efficient viral propagation in Huh7-PEDV-S cells at both MOIs. Taken together, these findings underscore the efficient replication capacity of rVSV∆G-PEDV-S in Huh7-PEDV-S cells, highlighting its potential for further virological research.

### 3.5. Vaccination of C57BL/6 Mice with rVSV∆G-PEDV-S Induced Neutralizing Antibodies Against PEDV

**Figure 5:**
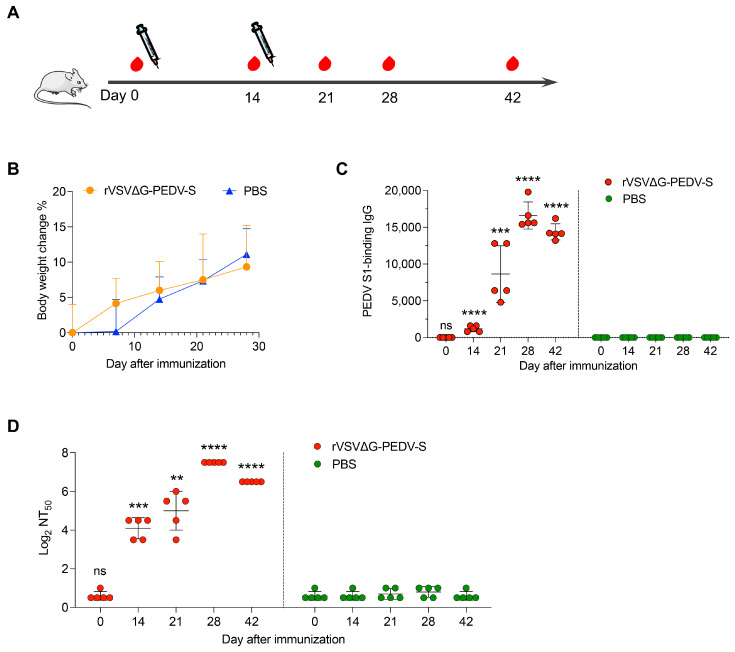
rVSVΔG-PEDV-S induces immune response against PEDV in BALB/c mice. (**A**) Mouse experimental schedule: Four-week-old BALB/c female mice (*n* = 5 for each group) were intramuscularly (IM) immunized with 1 × 10^8^ TCID_50_ of rVSV∆G-PEDV-S, followed by a booster immunization with the same dose 14 days later. (**B**) Changes in body weight of the vaccinated mice receiving PBS (*n* = 5) compared to those vaccinated with rVSV∆G-PEDV-S (*n* = 5) are presented. (**C**) Specific immunoglobulin G (IgG) antibodies binding to PEDV-S1 were quantified using enzyme-linked immunosorbent assay (ELISA). (**D**) Neutralizing antibody titers (NT_50_) against rPEDV SD-EGFP were determined through a neutralization assay and calculated using the Reed–Muench method. Statistical significance between the rVSVΔG-PEDV-S and PBS groups was assessed using a one-way ANOVA Kruskal–Wallis test. ns, not significant; **, *p* < 0.01; ***, *p* < 0.001; ****, *p* < 0.0001.

The rVSV-based vaccine platform has demonstrated efficacy in the development of vaccines against various pathogens, including the Ebola virus, SARS-CoV-2, and the Nipah virus, through the expression of the viral surface glycoprotein (GP) as the immunogenic antigen. To explore the PEDV-specific immunogenicity of rVSV∆G-PEDV-S in vivo, 4-week-old BALB/c female mice were inoculated with 10^8^ TCID_50_ of rVSV∆G-PEDV-S per mouse through the intramuscular routes, with PBS as the vaccination control. Immunization was boosted with the same dose 14 days after the initial immunization, and, notably, no adjuvants were used during the vaccination process. The animals’ weights were recorded daily, and serum samples were collected one day before both the prime and booster vaccinations (Figure 5A). Mice were then monitored weekly for weight loss or visible signs of disease. Remarkably, all mice vaccinated with the rVSV∆G-PEDV-S vaccine exhibited no adverse effects, remained healthy, and demonstrated gradual weight gain. Importantly, no significant changes in body weight were observed in the PBS control group compared to the group vaccinated with rVSV∆G-PEDV-S (Figure 5B), which indicates the safety of the rVSV∆G-PEDV-S vaccine. Serum samples were collected from all mice post-primary immunization and booster doses. The IgG titers against the PEDV S1 protein were evaluated using ELISA, while the neutralizing antibody titers against rPEDV-SD-EGFP infection were determined through fluorescence-reduction neutralization assays. The results revealed that PEDV S-specific IgG antibodies were detectable starting on day 14 after the primary immunization, and a notable increase in antibody titers to 1:8640 was observed on day 21 following the booster dose (Figure 5C). Furthermore, neutralizing antibody titers peaked at 1:181 on day 28 (Figure 5D) and remained robust through day 42. These findings highlighted that the rVSV∆G-PEDV S vaccine successfully induced a strong immune response against PEDV in adult BALB/c mice. The generation of effective neutralizing antibodies against PEDV demonstrates the potential of this vaccine to protect mice from PEDV infection.

## 4. Discussion

PEDV continues to be a significant challenge to swine farming globally, with outbreaks frequently leading to substantial economic losses. The ongoing emergence of highly virulent PEDV variants exacerbates the difficulty of controlling the disease. Current vaccines, which include inactivated and live-attenuated formulations, face limitations, such as suboptimal immunogenicity and safety concerns related to potential reversion to virulence. In light of these challenges, there is an urgent need for vaccines that can rapidly address emerging PEDV outbreaks, provide high immunogenicity, and ensure robust safety. The PEDV surface S glycoprotein plays critical roles in interactions with host cells and represents a major target for neutralizing antibodies. In this study, we developed and characterized a stable Huh7-PEDV-S cell line to generate pseudotyped replication-deficient rVSV particles that the PEDV S protein incorporated into the surface of rVSV. Stable expression of the S protein in Huh7 cells was successfully achieved through lentiviral vector-mediated transduction, antibiotic selection, and single-cell cloning. Western blot and immunofluorescence staining analysis confirmed robust S protein expression. This cell line is valuable not only for PEDV vaccine development but also as a research tool for understanding virus–host interactions and studying the mechanisms of PEDV entry and infection.

We generated a PEDV spike-pseudotyped rVSV∆G named rVSV-∆G-PEDV-S using the Huh7-PEDV-S cell line. The rVSV-∆G-PEDV-S was designed to lack the glycoprotein (G) gene, thereby rendering the virus defective for replication in normal tissue cells due to its inability to produce progeny virions. Instead, replication and propagation are achieved only in the Huh7-PEDV-S cell system, which provides the complete S protein in a complementary manner. This replication deficiency represents a significant safety advantage, as the virus cannot establish a propagating infection in vaccinated hosts. We successfully confirmed the incorporation of the PEDV S protein into the rVSV virions via Western blot assays. The resulting pseudovirus particles exhibited typical CPE in PEDV-susceptible cell lines, including Vero, LLC-PK1, and Huh7 cells, indicating efficient cell entry mediated by the S protein and underscoring the functional integrity of the pseudotyped particles.

An essential objective of this study was to evaluate the immunogenicity of rVSV-∆G-PEDV-S in vivo. Using a BALB/c mouse model, we demonstrated that two doses of the pseudotyped vaccine (administered intramuscularly) were sufficient to induce strong PEDV-specific humoral immune responses. The neutralizing antibody response was notable, peaking at day 28 post-primary immunization and demonstrating effective inhibition of PEDV infection in in vitro assays using fluorescence-reduction neutralization techniques. The recombinant rVSVΔG-PEDV-S virus demonstrates promising safety characteristics, making it a strong candidate for a PEDV vaccine. A previous study utilized a highly attenuated recombinant VSV as a vector to express the PEDV S protein [16]. However, that recombinant VSV retained both the G protein of VSV and the PEDV S protein. In contrast, the current study employs rVSVΔG-PEDV-S, a system that entirely lacks G protein expression. The VSV-G gene is recognized as the primary virulence determinant of VSV, and its deletion in this approach ensures a high safety profile, even at elevated doses. This significantly reduces the likelihood of adverse effects in animal models, addressing a key concern in vaccine development. The issue of recombination is a critical factor in RNA virus evolution, including in coronaviruses. Specifically for PEDV, recombinant strains with increased pathogenicity have been observed in the field due to genetic exchanges between attenuated vaccine strains and virulent field strains in the spike gene [31]. These events highlight the inherent safety challenges associated with live-attenuated vaccines and viral vector vaccines expressing the PEDV spike gene. Given the continued evolution of PEDV through recombination during outbreaks, the replication-defective nature of rVSVΔG-PEDV-S offers a particularly safe alternative for vaccine development. By not integrating the PEDV spike gene into the viral genome, this system prevents genetic recombination with field strains, addressing a significant safety concern. The unique attributes of rVSVΔG-PEDV-S, namely its replication-defective design and exclusion of the PEDV spike gene, underscore its potential as an innovative next-generation vaccine platform. This approach successfully combines efficacy with biosecurity, making it a highly promising candidate for combating PEDV.

Our study aligns with prior reports on rVSV-based vaccine platforms for other viral pathogens, including the Ebola virus, Crimean–Congo hemorrhagic fever virus, SARS-CoV-2, and Zika virus [21,22,23,24,25,45]. These platforms exhibit several advantages, including ease of production to high titers, an ability to elicit both humoral and cellular immunity, and compatibility with large-scale manufacturing processes. By leveraging many of these established benefits while addressing the specific need to target PEDV, we expand the versatility of the rVSV vaccine platform. Unlike conventional vaccines, the pseudotyped rVSV platform can rapidly adapt to incorporate new S proteins from emerging PEDV variants, offering a promising avenue for addressing viral evolution and antigenic drift.

Despite these promising results, several important questions remain. First, while the murine vaccination studies offer insights into immune responses elicited by rVSV-∆G-PEDV-S, swine models are necessary to confirm its efficacy, potency, and protective capabilities in the natural host. A critical next step will be evaluating the induction of mucosal immunity in swine, a key consideration for an enteric pathogen, such as PEDV. In addition, further studies will focus on the critical aspects necessary to validate the efficacy of the rVSVΔG-PEDV vaccine in pigs. These include evaluating the durability of both humoral and cellular immune responses to ensure long-term protection, optimizing dosage and vaccination schedules for practical application, identifying immunological correlates of protection, such as antibody titers or T-cell responses, conducting challenge studies to directly assess protection against clinical disease, and comparing the vaccine’s efficacy with existing PEDV vaccines to establish its relative advantages. These efforts will provide a comprehensive understanding of the vaccine’s potential in controlling PEDV outbreaks in swine, particularly in regions where the disease remains a significant challenge.

## 5. Conclusions

In this study, we developed and characterized a replication-deficient rVSV-based vaccine candidate for PEDV, which showed excellent safety and immunogenicity. The vaccine elicited strong PEDV-specific humoral responses, including high neutralizing antibody titers, in a mouse model, without any adverse effects. Its replication-deficient design ensures safety, while the stable incorporation of the S protein enables robust pseudovirus production and adaptability to emerging PEDV variants. These features position rVSV-∆G-PEDV-S as a promising and versatile vaccine candidate for PEDV control. Future studies in swine models are essential to confirm its protective efficacy and potential for controlling PEDV outbreaks in real-world settings, paving the way for broader applications in managing swine coronaviruses.

## Figures and Tables

**Table 1:** Oligo nucleotide sequences used in this study.

Oligo Name	Sequence (5′-3′)	Purpose
VSV-EcoRV_F	CATATGAAAAAAACTAACAGA	pVSV∆G
VSV-EcoRV_R1	ACTCGAGCCCGGGACGCGTAGG TGTCAAGGAAACAGATCGAT	pVSV∆G
VSV-EcoRV_R2	GTTCAAACATGAAGAATCTGTTGTGCA GGATTTGAACTCGAGCCCGGGACGCGTA	pVSV∆G
VSV-EcoRV_R3	AAGGCCTCTTTGAGCATGATATCAC AAGTTGATTTGGTTCAAACATGAAGAAT	pVSV∆G
qPCR-VSV-N_F	CAAATGATGCTTCCAGGCCA	Virus titer
qPCR-VSV-N_R	CAATGTCATCAGGCTGTCGG	Virus titer

**Table 2:** Experimental design of mouse immunization.

Group	Inoculum	Routes	Immunization Dose	Immunization Days
1	rVSV∆G-PEDV-S	IM	10^8^ TCID_50_/100 μL	D0, D14
2	PBS	IM	100 μL	D0, D14

## Data Availability

The data that support the findings of this study are available from the corresponding author upon reasonable request.
